# Clathrin light chain A facilitates small extracellular vesicle uptake to promote hepatocellular carcinoma progression

**DOI:** 10.1007/s12072-023-10562-5

**Published:** 2023-06-24

**Authors:** Yi Xu, Yue Yao, Liang Yu, Hiu Ling Fung, Alexander Hin Ning Tang, Irene Oi-Lin Ng, Melody Y. M. Wong, Chi-Ming Che, Jing Ping Yun, Yunfu Cui, Judy Wai Ping Yam

**Affiliations:** 1 Department of Pathology, School of Clinical Medicine, Li Ka Shing Faculty of Medicine, The University of Hong Kong, Queen Mary Hospital, 7/F Block T, Pokfulam, Hong Kong, China; 2 Department of Hepatopancreatobiliary Surgery, Second Affiliated Hospital of Harbin Medical University, Harbin, Heilongjiang People’s Republic of China; 3 Department of Endocrinology and Metabolism, Second Affiliated Hospital of Harbin Medical University, Harbin, Heilongjiang People’s Republic of China; 4 State Key Laboratory of Liver Research, The University of Hong Kong, Hong Kong, China; 5 Laboratory for Synthetic Chemistry and Chemical Biology Limited, Hong Kong Science Park, Hong Kong, China; 6 State Key Laboratory of Synthetic Chemistry, Department of Chemistry, The University of Hong Kong, Pokfulam Road, Hong Kong, China; 7 Department of Pathology, Sun Yat-Sen University Cancer Center, Guangzhou, Guangdong People’s Republic of China

**Keywords:** Hepatocellular carcinoma, Small extracellular vesicles, Clathrin light chain A, Endocytosis, Patient-derived xenografts, Pitstop 2, Capping actin protein gelsolin-like, Biomarkers

## Abstract

**Background:**

Endocytosis is a fundamental process for internalizing small extracellular vesicles (sEVs). The present study aimed to elucidate the role of clathrin light chain A (CLTA) in sEV uptake in hepatocellular carcinoma (HCC).

**Materials and methods:**

CLTA expression was analyzed by bioinformatics, quantitative PCR and immunohistochemistry. The clinical relevance of CLTA was analyzed by Fisher’s exact test, Kaplan–Meier analysis, and multivariate cox regression model. The functions of CLTA in sEV uptake and cancerous properties were examined by PKH67-sEV uptake, MTT, colony formation, and transwell assays. Mass spectrometry was used to identify the downstream effectors of CLTA. CLTA inhibitor, Pitstop 2, was tested in a mouse model of patient-derived xenografts (PDXs).

**Results:**

CLTA expression was higher in tumor tissues than in non-tumorous liver tissues and progressively increased from the early to late tumor stage. CLTA overexpression was associated with larger tumor size and poor prognosis in HCC. Cellular CLTA contributed to the sEV uptake, resulting in enhanced cancerous properties. Mechanistically, CLTA increases capping actin protein gelsolin-like (CAPG) expression to facilitate sEV uptake, thereby promoting the proliferation, motility, and invasiveness of HCC cells. What’s more, the CLTA inhibitor Pitstop 2 alone or in combination with sorafenib attenuated tumor growth in mice implanted with PDXs.

**Conclusions:**

The study reveals the role of CLTA in sEV uptake to promote HCC progression. Inhibition of CLTA and its mediated pathway illuminate a new therapeutic strategy for HCC patients.

**Supplementary Information:**

The online version contains supplementary material available at 10.1007/s12072-023-10562-5.

## Introduction

Liver cancer is a serious disease threatening human health all over the world, especially for East and Southeast Asian people [1]. Hepatocellular carcinoma (HCC), the most frequent neoplasm among all primary liver cancer, is currently the third leading cause of cancer related deaths worldwide [2]. Patients diagnosed at an advanced stage are only eligible for palliative treatments and the expected overall life expectancy is less than 1 year [3]. Therefore, it is particularly important to deeply investigate the pathological process of progression of HCC for the development of novel targeted therapies.

Small extracellular vesicles (sEVs), also known as exosomes, are a heterogeneous population of membrane vesicles of multiple origins, which are present in biological fluids and correlates with numerous physiological and pathological processes [4, 5]. The successful uptake of sEVs by cells is indispensable for cell‒cell communication via transfer of their cargos [6]. However, it remains unclear how HCC cells take up sEVs, which are crucial for advancing understanding of the tumor-microenvironment interactions of HCC. A recent study indicated that macropinocytosis and endocytosis are key processes that are responsible for recipient cells to take up sEVs [7]. Macropinocytosis is an sEV uptake approach that creates typical invaginated membrane ruffles to pinch off into the intracellular compartment. Na^+^/H^+^ exchanger may be essential in maintaining intracellular alkalization and favorable for micropinocytosis [8]. A variety of endocytic pathways have been proposed to regulate sEVs uptake by different cell types [9]. Elevated level of cholesterol combined with attachment of caveolin scaffolding domains to cell membrane and dynamin-2 activity enable assembly and expansion of caveolar endocytic vesicles [10]. Uptake of sEVs can be abrogated by blocking dynamin-2, indicating the role of caveolin-mediated endocytosis in sEVs internalization [11].

The clathrin-dependent pathway is another pivotal mechanism by which the cell membrane is rearranged to facilitate sEV uptake [7]. Clathrin light chain A (CLTA) is one of the three subunits of the light chain of clathrin. Together with heavy chains, clathrin light chains form clathrin as a structural component of cytoplasmic coated pits, which are associated with receptor-mediated endocytosis. A recent study indicated that CLTA could drive selective myosin VI recruitment to clathrin-coated pits under membrane tension [12]. It is also reported as an important factor responsible for membrane deformation and synaptic vesicle formation [13]. Depletion of CLTA also blocked the interaction of clathrin with the nucleation-promoting factor Wave complex to alter actin distribution [14].

However, the functions and mechanisms of CLTA in HCC remains unclear. In this study, we revealed the role of CLTA in sEV uptake by regulating the capping actin protein gelsolin-like (CAPG), thereby promoting HCC progression. In addition, frequent overexpression of CLTA was observed in HCC and correlates with dismal clinical characteristics. Moreover, CLTA inhibitor Pitstop 2 alone or in combination with sorafenib could inhibit patient-derived xenografts (PDXs) tumor growth.

## Materials and methods

### Human tissues

Clinical tissue specimens were used in this study. A tissue microarray (TMA) consisting of paired cases of tumor and adjacent nontumor liver tissues was constructed using tissue blocks provided by the Department of Pathology, Sun Yat-sen University Cancer Centre, China. The tissue samples used for RNA quantification was acquired from Queen Mary Hospital, Hong Kong. Approval for the use of human tissues was sought from the Institutional Review Board of The University of Hong Kong/Hospital Authority Hong Kong West Cluster (HKU/HA HKW IRB) and Sun Yat-sen University Cancer Centre. All study procedures involving human specimens were handled according to the relevant ethical regulations.

### Statistical analysis

All data presented in the study are the mean ± standard error of the mean (SEM). The procedures were repeated in triplicate and analyzed by Student’s *t* test or one-way ANOVA using GraphPad Prism 8.30 (GraphPad, Inc., La Jolla, CA, USA). Overall survival (OS) and disease-free survival (DFS) of the individuals were evaluated by Kaplan–Meier curves with the log-rank test. Fisher’s exact test was used to investigate the correlations between CLTA expression and clinical characteristics. Cox proportional hazards regression with multivariate analysis was applied to assess the independent prognostic factors for HCC patients. *p* < 0.05 was considered statistically significant.

Further detailed experimental procedures are described in the Supplementary Materials and Methods section.

## Results

### CLTA expression is upregulated in hepatocellular carcinoma

**Fig. 1:**
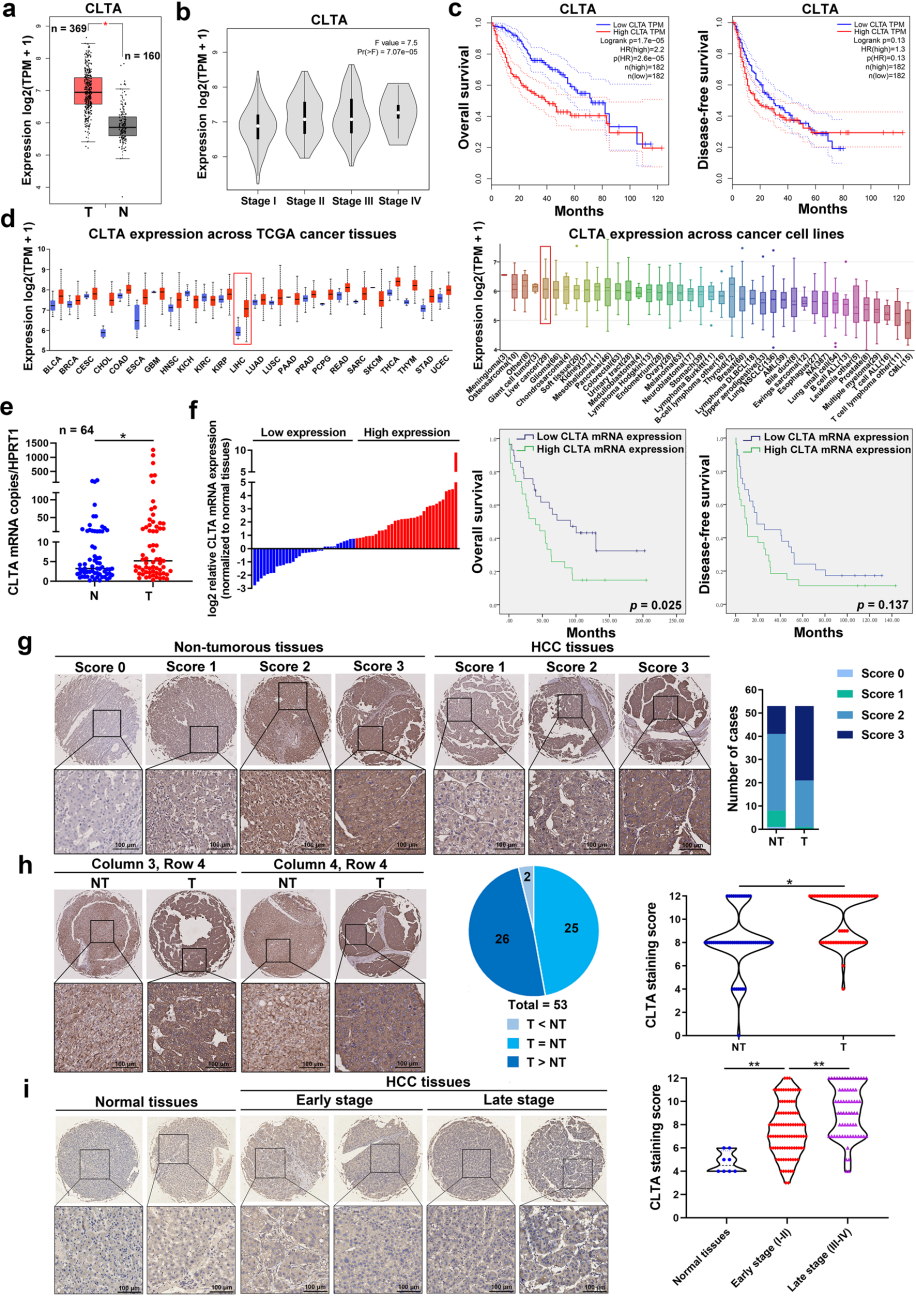
CLTA is frequently overexpressed in HCC. **A** Analysis of CLTA expression in tumor tissue (T, n = 369) and non-cancerous tissues (N, n = 160) using TCGA and GTEx datasets of liver cancer. **B** CLTA expression in tissues of HCC patients at different tumor stages. **C** Kaplan–Meier analysis of overall survival (*left*) and disease-free survival (*right*) of HCC patients with high and low CLTA expression based on the TCGA dataset. **D** CLTA expression across TCGA cancer tissues (*left*) and cancer cell lines (*right*). **E** qRT-PCR of CLTA mRNA expression in 64 pairs of in-house HCC tumor (T) and non-tumorous tissues (NT). **F** The in-house HCC cohort was divided into 2 groups of high and low CLTA expression using the median as a cutoff (*left*). Kaplan–Meier analysis of OS (*middle*) and DFS (*right*) in HCC patients according to CLTA expression. **G** Immunohistochemistry of CLTA was performed on TMA comprises paired T and NT tissues (n = 53). Representative images are shown (*left*), and the CLTA intensity score was analyzed (*right*). Scale bar, 100 μm. **H** Representative images of cases with CLTA overexpression in T (*left*). The pie chart illustrates the number of cases with overexpression, underexpression and no change in CLTA (*middle*). CLTA staining score of T and NT tissues (*right*). Scale bar, 100 μm. **I** Immunohistochemistry of CLTA was performed on TMA consisting of normal tissues (n = 8), early-stage (n = 98) and late-stage (n = 67) HCC tissues. Scale bar, 100 μm. *p < 0.05; **p < 0.01

Clathrin has three highly conserved light chain subunits, including CLTA, CLTB, and CLTC. The Cancer Genome Atlas (TCGA) and GTEx databases showed that CLTA was the only CLT subunit that was significantly upregulated in HCC tumor tissues (n = 369) compared to noncancerous liver tissues (n = 160) (Fig. 1A; Fig. S1A). Elevated expression of CLTA was positively correlated with the TNM stage of HCC (Fig. 1B; Fig. S1B). Although Kaplan–Meier analysis predicted a worse overall survival in HCC patients with high expression of CLTA, CLTB, or CLTC, the hazard ratio of CLTA (HR, 2.2) was higher than CLTB (HR, 1.5) and CLTC (HR, 1.8) (Fig. 1C; Fig. S1C-S1D). Across the TCGA cancer tissues, CLTA was elevated in most types of human cancers, among which HCC and cholangiocarcinoma were the most significantly upregulated. Additionally, CLTA was found to be overexpressed in liver cancer cells, which ranked 5th among the 40 cell types examined (Fig. 1D). In line with the expression of CLT subunits in clinical tissues, overexpression of CLTA was detected in all metastatic HCC cell lines compared to nonmetastatic cells and normal liver cells, while no correlation with the metastatic potential was found in CLTB and CLTC overexpressed cells (Fig. S1E). Therefore, we focused on the CLTA for study.

The expression of CLTA was further analyzed using an in-house cohort of 64 HCC cases. The overall expression of CLTA was significantly higher in tumor tissues than in paired noncancerous tissues (Fig. 1E). High CLTA expression was associated with larger tumor size (*p* = 0.042) and shorter overall survival (*p* = 0.025) but not disease-free survival (*p* = 0.137) (Fig. 1F; Table S4). While, the other characteristics such as gender (*p* = 1.000), age (*p* = 0.597), microsatilite (*p* = 0.781), liver cirrhosis (*p* = 0.414), number of tumors (*p* = 0.465), lymph node metastasis (*p* = 0.561), vein invasion (*p* = 0.275), TNM stage (*p* = 0.254), Edmondson-Steiner grade (*p* = 0.577), HBV infection (*p* = 0.414), HCV infection (*p* = 0.090), and serum AFP (*p* = 0.779) were not correlated with CLTA expression (Table S4). In addition, using multivariate analysis, high CLTA expression was identified as an independent prognostic factor for overall survival (*p* = 0.042) but not for disease-free survival of HCC patients (Table S5, S6).

Immunohistochemistry (IHC) staining of CLTA in 53 paired cases of HCC tissues and adjacent noncancerous tissues showed that 60.4% (32/53) of tumors were scored as strongly positive compared to 22.6% (12/53) of cases with strongly positive staining in non-tumorous tissues (Fig. 1G). CLTA was overexpressed in 49.06% (26/53) of cases (Fig. 1H). In another tissue microarray consists of normal liver tissues, early-stage HCC tissues and late-stage HCC tissues, we observed that CLTA immunoreactivity was remarkably higher in tumor tissues than in normal liver tissues and progressively increased from the early to late tumor stage (Fig. 1I). These findings suggest that high expression of CLTA is clinically relevant to HCC.

### CLTA promotes sEV uptake, cell proliferation, motility and invasiveness of HCC

**Fig. 2:**
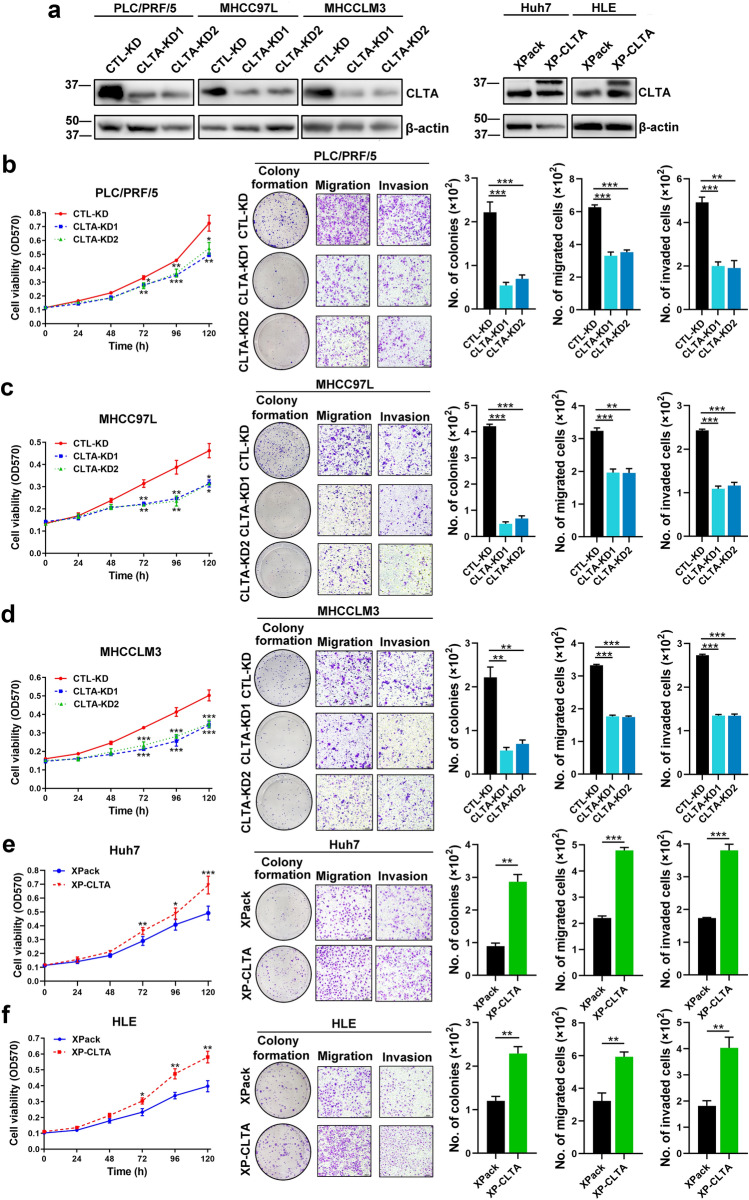
CLTA promotes HCC growth, migration and invasion. **A** Immunoblotting of CLTA in PLC/PRF/5, MHCC97L, MHCCLM3, Huh7 and HLE cells stably transfected with indicated vectors. **B** Cell viability, colony forming, cell migration and invasion capacities evaluated by MTT, colony formation, transwell migration and invasion assays in PLC/PRF/5 cells stably transfected with indicated vectors. Scale bar, 200 μm. **C** Cell viability, colony forming, cell migration and invasion capacities evaluated by MTT, colony formation, transwell migration and invasion assays in MHCC97L cells stably transfected with indicated vectors. Scale bar, 200 μm. **D** Cell viability, colony forming, cell migration and invasion capacities evaluated by MTT, colony formation, transwell migration and invasion assays in MHCCLM3 cells stably transfected with indicated vectors. Scale bar, 200 μm. **E** Cell viability, colony forming, cell migration and invasion capacities evaluated by MTT, colony formation, transwell migration and invasion assays in Huh7 cells stably transfected with indicated vectors. Scale bar, 200 μm. **F** Cell viability, colony forming, cell migration and invasion capacities evaluated by MTT, colony formation, transwell migration and invasion assays in HLE cells stably transfected with indicated vectors. Scale bar, 200 μm. Data are presented as the mean ± SEM. *p < 0.05; **p < 0.01; ***p < 0.001

**Fig. 3:**
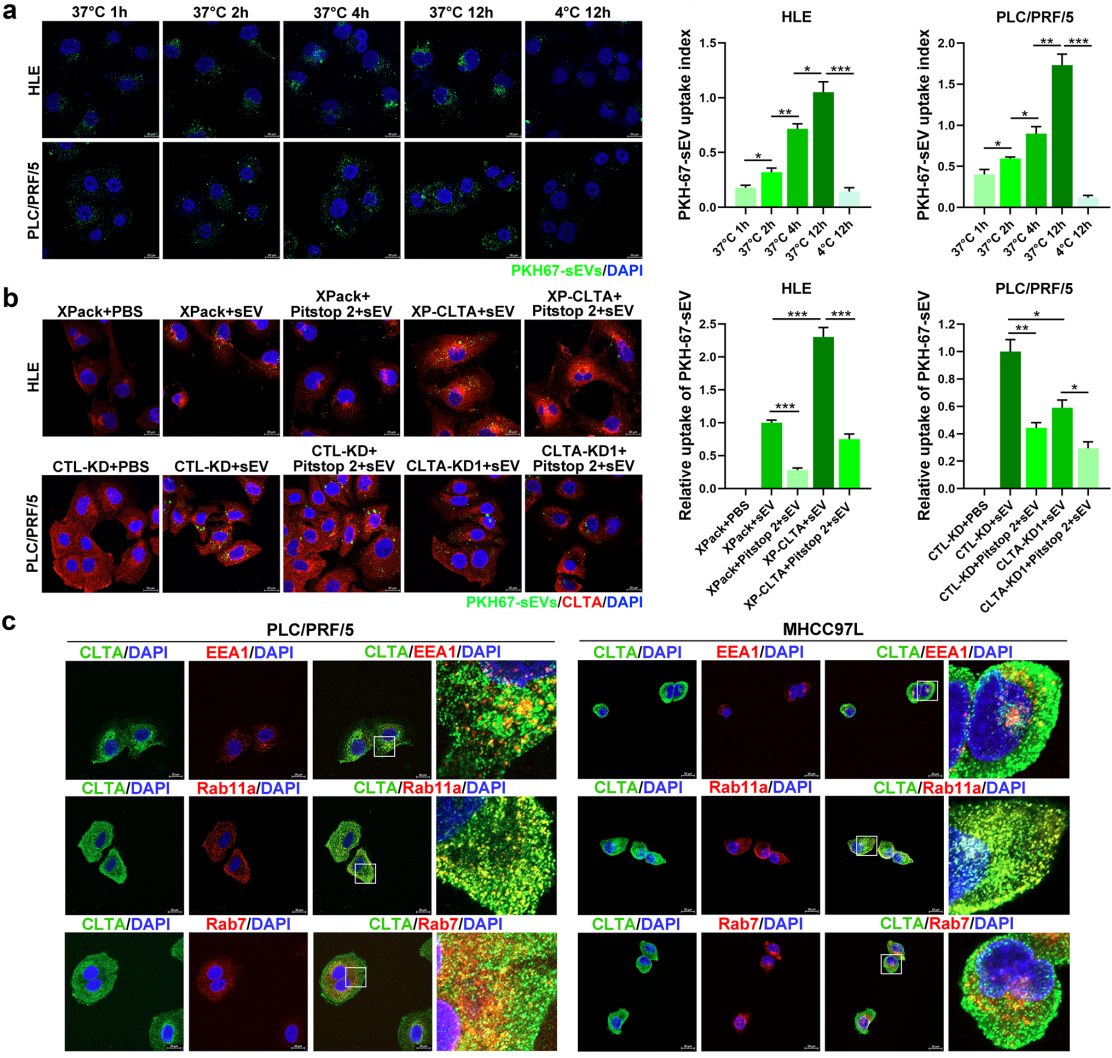
CLTA promotes sEV uptake. **A** HLE and PLC/PRF/5 cells were incubated with PKH67-labeled sEVs (green) for different durations and temperatures and observed by confocal microscopy. Scale bar, 20 μm. The intensity of the signal was analyzed. **B** Internalization of PHK67-labeled sEVs was analyzed in HLE XPack and XP-CLTA cells with or without the addition of Pitstop 2 (*upper panel*). A similar analysis was performed using PLC/PRF/5 CTL-KD and CLTA-KD cells (*lower panel*). Scale bar, 20 μm. The intensity of the signal was analyzed. **C** Immunofluorescence of colocalization of CLTA and EEA1/Rab11a/Rab7 in PLC/PRF/5 (*left*) and MHCC97L (*right*) cells. Scale bar, 20 μm. Data are presented as the mean ± SEM. *p < 0.05; **p < 0.01; ***p < 0.001

Functionally, reduction of CLTA in MHCCLM3, MHCC97L and PLC/PRF/5 cells showed a marked decrease in cell viability, colony formation ability, mobility and invasiveness (Fig. 2A–D). The effect of CLTA was corroborated with its enhancing effect in the viability and cancerous properties of Huh7 and HLE cells (Fig. 2A, E, F). Previous studies have demonstrated that the uptake of sEVs is mediated by clathrin-mediated endocytosis [15], which raises the possibility that CLTA might confer its oncogenic properties via mediation of sEV uptake. HLE and PLC/PRF/5 cells were incubated with PKH67-labeled sEVs at 37 °C. The amount of sEVs taken up by both cells increased with the incubation time. When the cells were incubated with sEVs at 4 °C, their ability of sEV uptake was significantly reduced, which implied that sEVs uptake is energy dependent and excluded the possibility of passive diffusion (Fig. 3A).

To answer whether CLTA is involved in the uptake of sEV, we extended our investigation by treating HLE CLTA-overexpressing (XP-CLTA) and PLC/PRF/5 CLTA knockdown (CLTA-KD) cells with PKH67-labeled sEVs. Overexpression of CLTA in HLE cells restored the sEV uptake tendency. Conversely, a reduction of CLTA in PLC/PRF/5 cells significantly hampered the ability of sEV uptake. The efficiency of sEV uptake was compromised after the addition of Pitstop 2, an inhibitor of clathrin, which implied a role of CLTA in clathrin-mediated sEV internalization in HCC (Fig. 3B; Fig. S2A–S2D). Immunofluorescence staining showed that CLTA was extensively colocalized with Rab11a (a recycling endosomal marker) and partially within the scope of EEA1 (an early endosomal marker) and Rab7 (a late endosomal marker) in HCC cells (Fig. 3C). The extensive distribution of CLTA with components of the endocytic pathway suggested its role in cellular endocytosis.

### CAPG is the functional component of CLTA in HCC

**Fig. 4:**
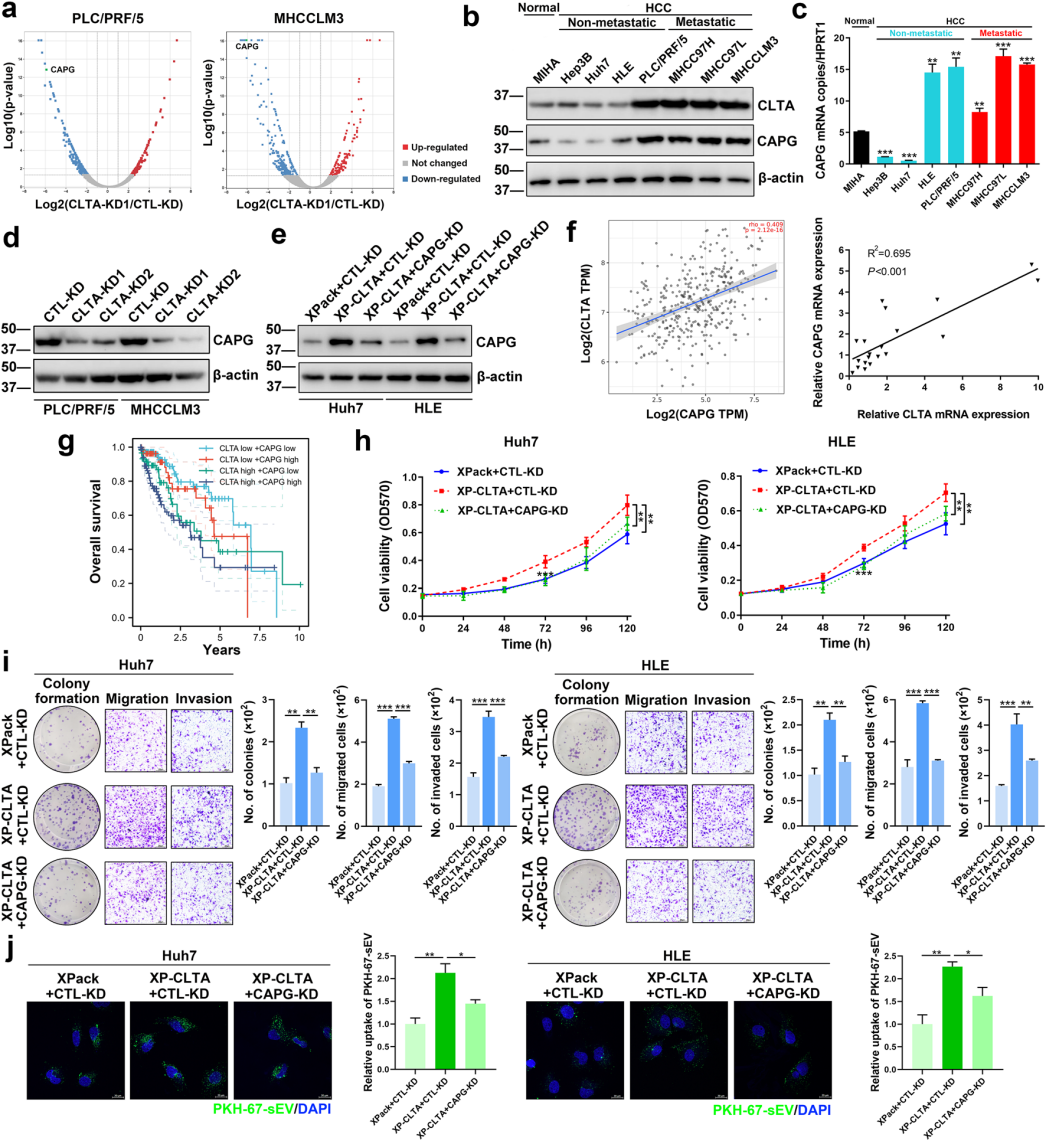
CAPG is a downstream regulator of CLTA in HCC. **A** Volcano plots of proteins that were significantly modulated by at least twofold in PLC/PRF/5 CLTA-KD (*left*) and MHCCLM3 CLTA-KD (*right*) cells compared to proteins in their respective control CTL-KD cells. Immunoblotting **B** and qRT-PCR analysis **C** of CLTA and CAPG expression in cell lines. **D** Immunoblotting of CAPG in PLC/PRF/5 and MHCCLM3 CLTA-KD and CLTA-KD cells. **E** Huh7 and HLE XPack and XP-CLTA cells transiently transfected with CTL-KD and CAPG-KD plasmids were analyzed for CAPG expression by immunoblotting. **F** Pearson’s correlation analysis of CLTA and CAPG expression using the TCGA dataset of liver cancer (*left*) and an in-house cohort of HCC samples (*right*). **G** Kaplan–Meier analysis of the overall survival of HCC patients according to CLTA and CAPG expression. Huh7 (*left*) and HLE (*right*) XPack and XP-CLTA cells transfected with CTL-KD and CAPG-KD vectors were subjected to cell viability analysis **H** and colony formation, migration and invasion assays **I**. **J** The ability of the indicated cells to take up PKH67-labeled sEVs was analyzed. The intensity of the signal was plotted. Scale bar, 20 μm. Data are presented as the mean ± SEM. *p < 0.05; **p < 0.01; ***p < 0.001

To identify the downstream regulators of CLTA-mediated HCC development, mass spectrometry protein analysis was performed on PLC/PRF/5 and MHCC97L CTL-KD and CLTA-KD cells. Among all differentially expressed proteins, we observed that CAPG was significantly downregulated in both CLTA-KD cell lines (Fig. 4A). A similar trend of CAPG and CLTA expression in HCC cells was revealed by immunoblotting and qRT-PCR, notably for cells with high metastatic potential (Fig. 4B, C). CLTA knockdown in PLC/PRF/5 and MHCCLM3 cells strikingly inhibited the expression of CAPG, which complied with the CAPG expression profile screened by mass spectrometry (Fig. 4D). In contrast, Huh7 and HLE XP-CLTA cells showed enhanced CAPG expression which was suppressed by CAPG knockdown (Fig. 4E). The causal relationship between CLTA and CAPG was further observed in the TCGA database of liver cancer and in the in-house cohort of HCC samples, in which their expression was positively correlated (Fig. 4F). Furthermore, patients with overexpression of both CLTA and CAPG had the worst overall survival compared to patients with overexpression of either CLTA or CAPG (Fig. 4G).

Endocytosis involves reshaping of the membrane, which is mediated by the concerted efforts of endocytic proteins and actin cytoskeletal proteins [16]. CAPG is an actin-regulatory protein involved in cytoskeleton remodeling [17], suggesting the possibility of its involvement in CLTA-mediated uptake of sEVs and its oncogenic effect on HCC cells. It was shown that CAPG knockdown hampered CLTA-induced HCC cell viability, colony formation ability, migration, and invasiveness (Fig. 4H–I). The uptake of sEVs by XP-CLTA cells was also compromised when CAPG was suppressed (Fig. 4J). Taken together, these data revealed that CLTA and CAPG are functionally related and physiologically relevant in HCC.

### Blockade of CLTA using inhibitor suppresses the development of HCC patient-derived xenografts

**Fig. 5:**
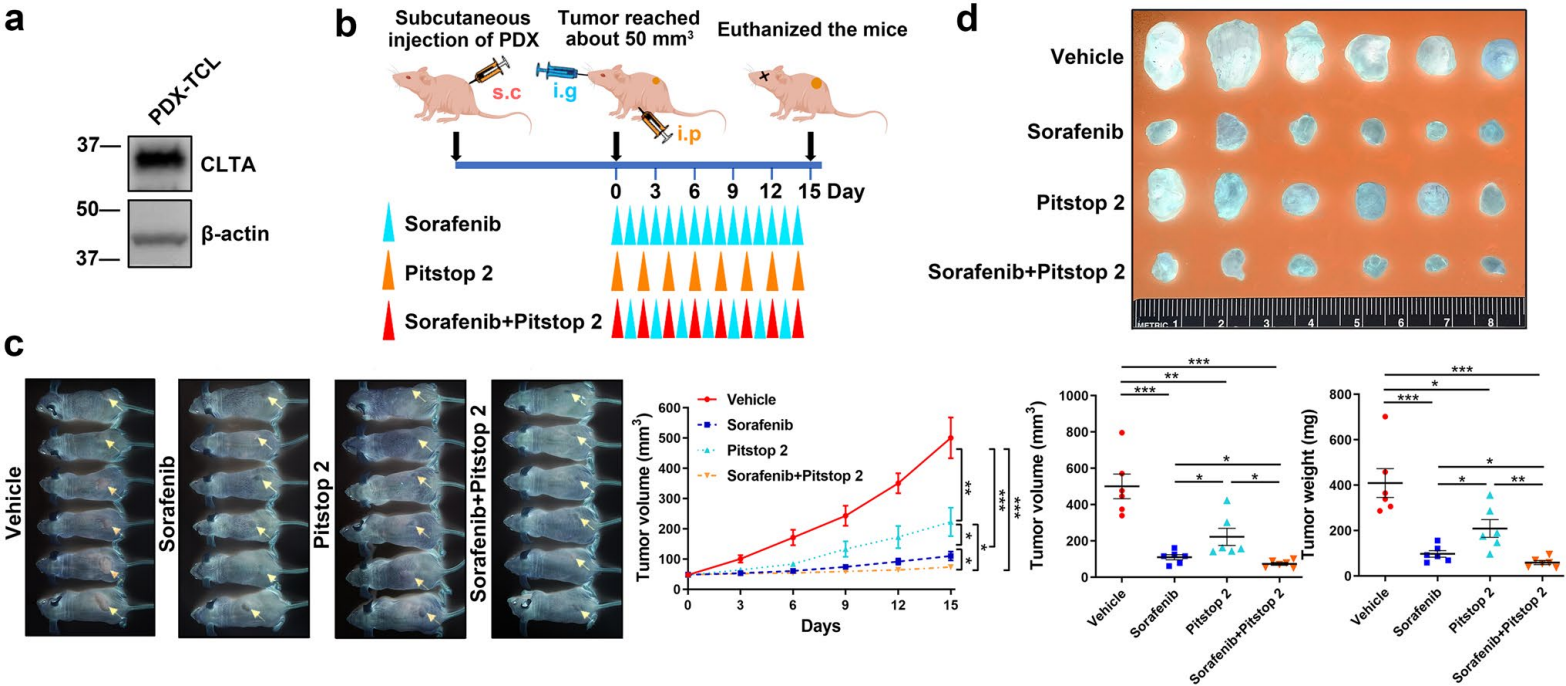
Blockade of CLTA using inhibitor suppresses the development of HCC patient-derived xenografts. **A** Immunoblotting of CLTA in total cell lysates from HCC PDXs. **B** The diagram illustrates the treatment regimen of sorafenib and Pitstop 2 administered to mice subcutaneously implanted with PDXs. **C** Image of mice at the end of the experiment (*left*). Tumor size was measured regularly and plotted (*right*). **D** The tumors were harvested at the end of the experiment. The volume and weight of the tumors were determined. Data are presented as the mean ± SEM. *p < 0.05; **p < 0.01; ***p < 0.001

This study revealed the role of cellular CLTA in driving HCC progression. Here, a mouse model of a subcutaneous HCC PDX that expresses CLTA was employed to test the therapeutic efficacy of pharmacological inhibition of cellular CLTA using Pitstop 2. CLTA could be detected in the total cell lysates of PDX tumor, implicating the physiological relevance of this model to study the function of CLTA (Fig. 5A). Pitstop 2 and sorafenib, the first-line treatment for advanced unresectable HCC, alone or in combination, were administered to mice implanted with PDXs (Fig. 5B). Both Pitstop 2 and sorafenib significantly inhibited tumor development and resulted in smaller tumors compared to those formed in untreated mice. Combined treatment showed an enhanced inhibitory effect compared to treatment using a single agent (Fig. 5C, D).

## Discussion

Clathrin functions as the main structural component of the lattice-type cytoplasmic face of coated pits to promote sEV uptake [18]. It is a protein complex consisting of heavy and light chains. It has been reported that caveolin-1, flotillin-1, RhoA, Rac1 and Pak1, rather than the clathrin heavy chain, are responsible for sEV uptake [19]. In such a scenario, it would stand to reason that clathrin light chain subunits play a key role in sEV internalization.

In this study, 3 members of the clathrin light chain family were included to evaluate their expression profile in HCC. CLTA was identified to be the most elevated in HCC cells, especially for those metastatic cells. However, CLTB and CLTC failed to show the similar trend. Considering our unpublished data showed highest sEV uptake potential, we focused our attention on CLTA. Subsequent functional assays indicated their ability to take up sEVs and its oncogenic role in promoting HCC progression and aggressiveness. Our findings revealed that CLTA was extensively colocalized with Rab11a (a recycling endosomal marker) and partially within the regions of EEA1 (an early endosomal marker) and Rab7 (a late endosomal marker) in HCC cells. The evidence of its extensive distributions in different phases of endosomes implicates the multifaceted involvement of CLTA in cellular endocytosis, intracellular transport, and endosome degradation.

To delineate how CLTA regulates sEV uptake, we performed proteomic profiling of cells transfected with CTL-KD and CLTA-KD1 plasmids. CAPG was identified as a downstream effector of CLTA. CAPG has been reported to be upregulated in various cancer types, such as ovarian cancer [20], bladder cancer [21], colorectal cancer [22], and glioma [23]. Overexpression of CAPG was statistically correlated with poor survival, lymph node metastasis and advanced tumor stage [24]. In both the TCGA database of liver cancer and the in-house cohort of HCC, we observed a positive correlation between CLTA and CAPG expression. The physiological relevance of their expression is congruent with their causal relationship observed in HCC cells, in which both are highly expressed in metastatic HCC cells.

Functionally, CAPG is an actin-regulatory protein that is able to reversibly block the barbed ends of F-actin filaments in a Ca^2+^ and phosphoinositide-regulated manner. By capping the barbed ends of actin filaments, the encoded protein contributes to the control of actin-based motility [25]. CAPG is required for receptor-mediated ruffling [26]. This kind of cytoskeleton remodeling function of CAPG may help CLTA endocytose sEVs. Here, we revealed the functions of CAPG, together with CLTA, in regulating the internalization of sEVs by cancer cells.

It is noted that the expression level of CLTA is not correlated with HBV or HCV infection based on our analysis. However, it is worth further investigation because the enrolled patients in our study are limited. We found a positive correlation between CLTA expression level and HCC tumor size. We speculate the higher level of CLTA allows the tumor cells to endocytose more nutrient substance including sEVs in tumor microenvironment, thereby facilitating the tumor growth. A recent study using single-cell RNA sequencing method identified a three-gene signature (CLTA, TALDO1 and CSTB) as an unfavorable prognosis for HCC patients [27]. In the present study, elevated CLTA was associated with larger tumor size and shorter overall survival and may be regarded as an independent prognostic factor for HCC patients. Moreover, a worse overall survival was identified in patients with high expression of both CAPG and CLTA than in those with either high CAPG or CLTA expression. It is not surprising that low expression of both CLTA and CAPG in HCC tissues predicts the best overall survival among the four subgroups. Nevertheless, this study still has some limitations. A larger cohort of patients should be recruited to validate the clinical significance of CLTA in the future study.

Sorafenib is indicated as a first-line systemic agent for unresectable and advanced HCC [28]. However, sorafenib has modest effect on tumor shrinkage. What’s worse, many patients are quite refractory to sorafenib after long-term use [29]. Emerging evidence indicated tyrosine kinase inhibitor (TKI), regorafenib, as a potential treatment option in patients with unresectable HCC who had previously failed first-line treatment with sorafenib [30]. In addition, current clinical practice also demonstrated the synergistic antitumor efficacy of immune checkpoint inhibitors (ICI) with TKI [31]. Nevertheless, it will be enhanced in the future with different available drugs and a better understanding of the synergy will hopefully help us to tackle down HCC lethality. In this study, we propose whether CLTA inhibitor could synergy with sorafenib to inhibit HCC PDXs progression. Indeed, the blockade of sEV uptake by Pitstop 2 could effectively inhibit the growth of HCC PDXs that express CLTA. When combined with sorafenib, Pitstop 2 further increased the therapeutic effect in inhibiting PDXs tumor growth. Therefore, the blockade of CLTA could be a way to improve the current therapeutics for HCC treatment.

In conclusion, the present study demonstrated the role of CLTA in HCC progression in mediating sEV uptake via CAPG. Additionally, frequent overexpression of CLTA was observed in HCC and correlates with worse overall survival. This study also provides insights into a new therapeutic strategy by inhibiting CLTA and blocking its mediated effect on sEV uptake.

### Supplementary Information

Below is the link to the electronic supplementary material.

Supplementary file1 (DOCX 535 KB)

## Data Availability

The data that support the findings of this study are available from the corresponding author, upon reasonable request.
