# Supplementary material for: Clathrin light chain A facilitates small extracellular vesicle uptake to promote hepatocellular carcinoma progression
Source: Hepatol Int. 2023 Jun 24;17(6):1490–9. doi: 10.1007/s12072-023-10562-5 (PMC10660914; doi:10.1007/s12072-023-10562-5)
Supplement: Supplementary file 1 — Supplementary file1 (DOCX 535 KB) [file 12072_2023_10562_MOESM1_ESM.docx]

**Clathrin light chain A facilitates small extracellular vesicle uptake to promote hepatocellular carcinoma progression**

Yi Xu^1,2,†^, Yue Yao^1,3,†^, Liang Yu^1,2,†^, Hiu Ling Fung^1^, Alexander Hin Ning Tang^1^, Irene Oi-Lin Ng^1,4^, Melody YM Wong^5^, Chi-Ming Che^5,6^, Jing Ping Yun^7^, Yunfu Cui^2^, Judy Wai Ping Yam^1,4,^*

**Table of contents**

**Supplementary Materials and Methods**.................................................................................2

**Supplementary Figures**..........................................................................................................7

Fig. S1.......................................................................................................................................7

Fig. S2.......................................................................................................................................8

**Supplementary Tables**............................................................................................................9

Table S1.......................................................................................................................................9

Table S2.....................................................................................................................................10

Table S3.....................................................................................................................................11

Table S4.....................................................................................................................................12

Table S5.....................................................................................................................................13

Table S6.....................................................................................................................................14

**Supplementary References**....................................................................................................15

**Supplementary Materials and Methods**

***Cell culture***

Human immortalized normal liver cell line MIHA was a gift from Jayanta Roy-Chowdhury (Albert Einstein College of Medicine, New York) [1]. A total of seven HCC cell lines (PLC/PRF/5, Hep3B, Huh7, HLE, MHCC97L, MHCC97H, and MHCCLM3) was used in this study. Non-metastatic HCC cell lines among that, Hep3B and PLC/PRF/5 were provided by American Type Culture Collection (ATCC), Huh7 and HLE were obtained from Japanese Collection of Research Bioresources (JCRB, Japan). Human metastatic HCC cell lines including MHCC97L, MHCC97H and MHCCLM3 were provided by Cancer Institute (Fudan University, China). 293FT was obtained from ATCC. All the cells above were cultured according to the recommendations of protocol and were tested routinely to avoid mycoplasma contamination. And HCC cells were identified by short tandem repeat profiling. The detailed description is available in **Table S1**.

***Construction of vectors and establishment of stable clones***

XPack™ Exosome Protein Engineering Technology (System Biosciences) was utilized as the recommendations of protocol to generate plasmids to express CLTA in sEVs (XP-CLTA). The CLTA sequence was amplified via PCR utilizing MHCC97L cDNA as a template and primers that surround the CLTA sequence. The empty XP-MCS-EF1𝛼-Puro vector was ultilized to create a vector control clone (XPack) as control. The sequence was verified by Sanger sequencing. Similarly, MISSION shRNA Plasmid (Sigma-Aldrich) was utilized to generate CLTA knockdown stable clones (CLTA-KD1 and CLTA-KD2) and MISSION^TM^ nontarget shRNA control vector (Sigma-Aldrich) was used to generate the Nontarget control clone (CTL-KD). The detailed sequences are available in (**Table S2**).

To establish stable clones, Lenti-PacTM HIV Expression Packaging Kit (#LT001, GeneCopoeia) was utilized to transfect plasmid into HEK293FT. After 16 hours, Titerboost reagent (#LT001, GeneCopoeia Inc.) was utilized to enhance virus production. 48 hours after transfection, polybrene (Sigma-Aldrich) and Viral supernatant were used to transduct HCC cells which were planted and allowed in a 6-well plate previously. 24 hours later, stable clones were selected applying Puromycin or Blasticidin (Gibco).

***sEV uptake assay***

The sEVs uptake kit PKH67 (Sigma-Aldrich, #PKH67GL) was used to label the sEVs fluorescently. After that, sEVs was washed with phosphate-buffered saline and gathered by ultracentrifugation for further operations. 5 × 10^4^ cells were planted on each coverslip and were allowed to adherend overnight. Then the cells were subjected to treatment of 10 μg sEVs mentioned above for different time duration and temperature, respectively. After that, cells on coverslip were treated with 4% formaldehyde in PBS for fixation and recorded as photos using laser scanning confocal microscopy. The data was analyzed by ZEN Blue Software.

***Cell viability assessment***

To examine cell viability, cells were plated in 96-well plates at the concentration of 1000 cells per well, allowed to adhere overnight. These cells were treated with 100 μl of DMEM medium with 0.5 mg/ml MTT at 37 °C after incubation for 24, 48, 72, 96, or 120 h, respectively. After DMSO treatment, the quantity of formazan crystals was then detected by recording absorbance at 570 nm using a microplate reader. Cell viability curves were plotted accordingly.

***Colony formation assay***

Cell colony formation was measured by a colony formation assay. 1000 cells of each groups were seeded in 6-well plates. Then, cells were cultured at 37℃ in DMEM containing 10% FBS for 12-14 days. After that, the wells were washed gently with phosphate-buffered saline and fixed with paraformaldehyde. After being stained with 1% crystal violet, the colonies were photographed and counted.

***Cell migration and invasion assays***

In cell invasion assays, Matrigel Basement Membrane Matrix (BD Bioscience) was applied to form a coat prior to cell seeding. Besides the above, for both assays, cells suspended in serum-free medium were seeded at a same density in the upper chamber, while a full medium with chemoattractant in lower chamber. After incubation for 16 hours, the cells on upper surface were removed and the cells on lower side were fixed with formalin. After staining with crystal violet, photography was conducted applying a microscope, following by cell counting.

***Quantitative real-time PCR (qRT-PCR)***

The total RNA from cells or tissues was extracted by applying the RNeasy Mini Kit (Qiagen) according to the manufacturer's instructions. To get complementary DNA, SuperScriptTM VILO cDNA synthesis kit was utilized to perform the reverse transcription. After that, cDNA was mixed with SYBR Green PCR Master Mix (Applied Biosystems, Roche Life Science) and corresponding primers following real-time PCR through the LightCycler® 480 System. The expression of each genes was evaluated relative to HPRT1 level and was quantified applying the comparative cycle threshold 2^-ΔΔCt^.

***Western blotting***

Total cell lysates of 20 μg were separated on 10% SDS-PAGE (Bio-Rad) and then transferred to polyvinylidene difluoride membranes (Amersham). The membranes were blocked using 5% milk/TBS-T (0.1% Tween-20) and subjected to incubate with primary antibody overnight. The membranes were washed using TBS-T prior to incubation with corresponding secondary antibodies for 2 hours. After another washing step, the intensities of the proteins were detected applying ECL^TM^ Western Blotting Detection Reagents. The images were collected and analyzed by ImageJ software. The detail information on antibodies were listed in **Table S3.**

***Co-treatment of Pitstop 2 with sorafenib in PDXs model***

BALB/cAnN-nu mice were subjected to subcutaneous injection with cells from patient-derived tumor xenografts suspended in PBS. They were divided into four groups for different treatment once the subcutaneous tumors reach approximately 50 mm^3^ ± 10%, including vehicle group (DMSO in PBS, 100 μl/day; by oral gavage), Pitstop 2 group (0.15 mg/2 days; by intraperitoneal injection), sorafenib (30 mg/kg/day; by oral gavage), and co-treatment of Sorafinib + Pitstop 2 group. The tumor’s length and width were measured routinely to track its growth. The formula used to determine the tumor volume is: 0.5 × Length × Width^2^. 15 days after treatment, the tumors were harvested after mice were sacrificed. The weight and volume of tumors were recorded and analyzed.

***Mass spectrometry***

Proteins from samples of sEVs or cells were extracted by bead beating with a Precellys homogenizer after the samples were diluted in 2M urea and 50mM Tri-Ethyl ammonium bicarbonate lysis buffer. Then samples were subjected to centrifugation at 14,000 g for 30 minutes followed by measurement of the proteins using BCA method. The proteins were extracted by bead beating with the Precellys homogenizer using samples of sEVs or cells that had been diluted in 2M urea and 50mM Tri-Ethyl ammonium bicarbonate lysis buffer. Overnight at -20°C, the samples were incubated with 600 μl of pre-cooled acetone. The materials were then digested by trypsin over an overnight period at 37°C. Following LC-MS/MS analysis, the tryptic peptide was dried with a speed vacuum and desalted with ZipTips. Using a nanoLC system connected to an Orbitrap Fusion Tribid Lumos, isolated peptides were analyzed (Thermo). Using a commercial C18 column with 1.9 μm particle size (75 μm inner diameter × 50 cm length), peptides were extracted. A linear gradient of 5-80% Buffer B (80% ACN in formic acid) was used to separate the mixture for 150 mins. With capillaries heated to 300 degrees Celsius, mass spectrometers were operated in positive-ion modes. For the MS scan, a maximum injection period of 50 ms and an automated gain control goal of 2 × 10^6^ were employed. The scan range was 350–1500 m/z with a resolution of 120,000. The mass spectrometer was performed in data-dependent acquisition mode using a top 10 approach. Spectra with a resolution of 30000 MS2 were generated using an AGC target of 1× 10^5^, a maximum ion injection time (IT) of 100 ms, 1.6 m/z isolation width, and a normalized collisional energy of 30. Precursor ions that were fragmentation-selected and their isotopes were actively eliminated for 40 s. The dataset identification for the mass spectrometry proteomics data was PASS02784, and it was uploaded to PeptideAtlas [2].

***Immunohistochemistry***

Immunohistochemistry was performed on a tissue microarray of 53 cases of paired HCC tissues and adjacent non-tumorous tissues provided by the Department of Pathology, Sun Yat-sen University Cancer Centre. A tissue microarray comprises 8 normal liver tissues, 98 early-stage, and 67 late-stage HCC tissues purchased from Servicebio, Wuhan, China. Samples from patients were fixed with formalin and embedded with paraffin, followed by cutting as a thickness of 5 μm. The sections were deparaffinized in xylene. After that, samples were rehydrated in descending alcohol gradients followed by antigen retrieval and endogenous peroxidase inhibition. The prepared samples were then subjected to primary CLTA antibody incubation at 4°C overnight, followed by anti-rabbit secondary antibody (Dako). Hematoxylin and eosin (H&E) were utilized to counterstained the section. NanoZoomer Digital Pathology System (Hamamatsu) was applied to generate high-quality digital images. The expression of CLTA was analyzed by pathologists, and each core of tissue microarray was scored as 0 for negative, 1 for weak positive, 2 for moderate positive and 3 for strong positive to quantify the intensity. The percentage of positive-stained cells was individually scored as 0 for lower than <5%, 1 for 5%-25%, 2 for 25%-50%, 3 for 50%-75%, and 4 for more than 75%. The CLTA staining score was acquired from the score of intensity multiplied by the score of the percentage of positive-stained cells. The CLTA staining score was in the range from 0 to 12.

***Immunofluorescence staining***

Immunofluorescence staining was conducted at room temperature. Cells were planted on coverslips and subjected to culture in an incubator until they adhered well. Then, cells were fixed using paraformaldehyde followed by permeabilization with 0.1% Triton-X-100. After that, cells were blocked with 3% BSA in 1% Triton/PBS for 1 hour. Cells were incubated with primary antibody for 3 hours and were washed utilizing 1% Triton/PBS for 3 times. Then cells were incubated with secondary antibody for 1 hour followed by nucleus-staining with DAPI for 20 minutes in the dark. Coverslips were mounted to glass slides carefully using Vectashield anti-fade mounting medium. Images were acquired using a confocal microscopy.

***Bioinformatics analysis***

Gene Expression Profiling Interactive Analysis, version 2.0 (GEPIA2.0) [3] based on TCGA and genotype-tissue expression datasets (GTEx) was utilized to analyze the expression profiles of CLTA between the HCC tumor tissues and non-tumorous tissues. The screening criteria in GEPIA2.0 were as follows: *p* < 0.05 and the cutoff of Log_2_(Fold change) was 0.1. In addition, exploration of the relationship between CLTA levels and clinical TNM stages, survival analysis, and pan-cancer analysis were also performed using GEPIA. Correlation between CLTA and CAPG expression was estimated by TIMER2.0 database [4]. For multiple-group survival analysis, the follow-up information obtained from clinical data was downloaded from Cancer Genome Atlas Liver Hepatocellular Carcinoma (TCGA-LIHC) dataset (https://www.genome.gov/Funded-Programs-Projects/Cancer-Genome-Atlas) using R-package, survminer R package (0.4.9 version) and survival R package (3.2-10 version) were used to visualize and analyze the data.

**Supplementary Figures**


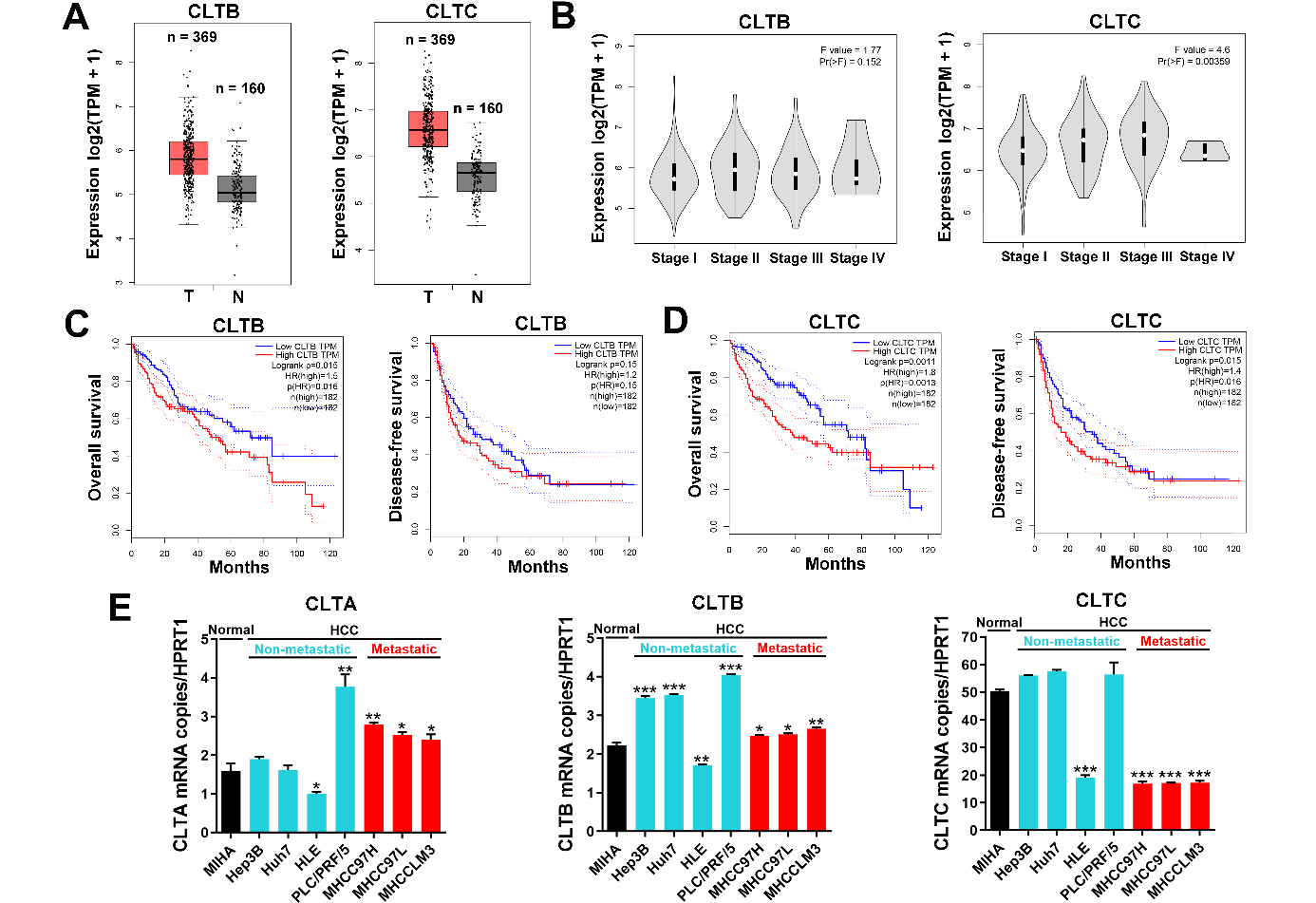


**Fig. S1. Expression profile of clathrin light chain family.** (A) CLTB and CLTC expression in HCC tumor (T, n = 369) and non-tumorous tissues (N, n = 160) analyzed by TCGA and GTEx datasets. (B) CLTB and CLTC expression in tissues from HCC patients with TNM stage I, II, III, and IV. (C) Kaplan-Meier analysis of OS (*left*) and DFS (*right*) in HCC patients according to CLTB expression by TCGA dataset. (D) Kaplan-Meier analysis of OS (*left*) and DFS (*right*) in HCC patients according to CLTC expression by TCGA dataset. (E) qRT-PCR of CLTA (*left*), CLTB (*middle*) and CLTC (*right*) mRNA expression in HCC cells and MIHA. Data are presented as the mean ± SEM. **p* < 0.05; ***p* < 0.01; ****p* < 0.001.


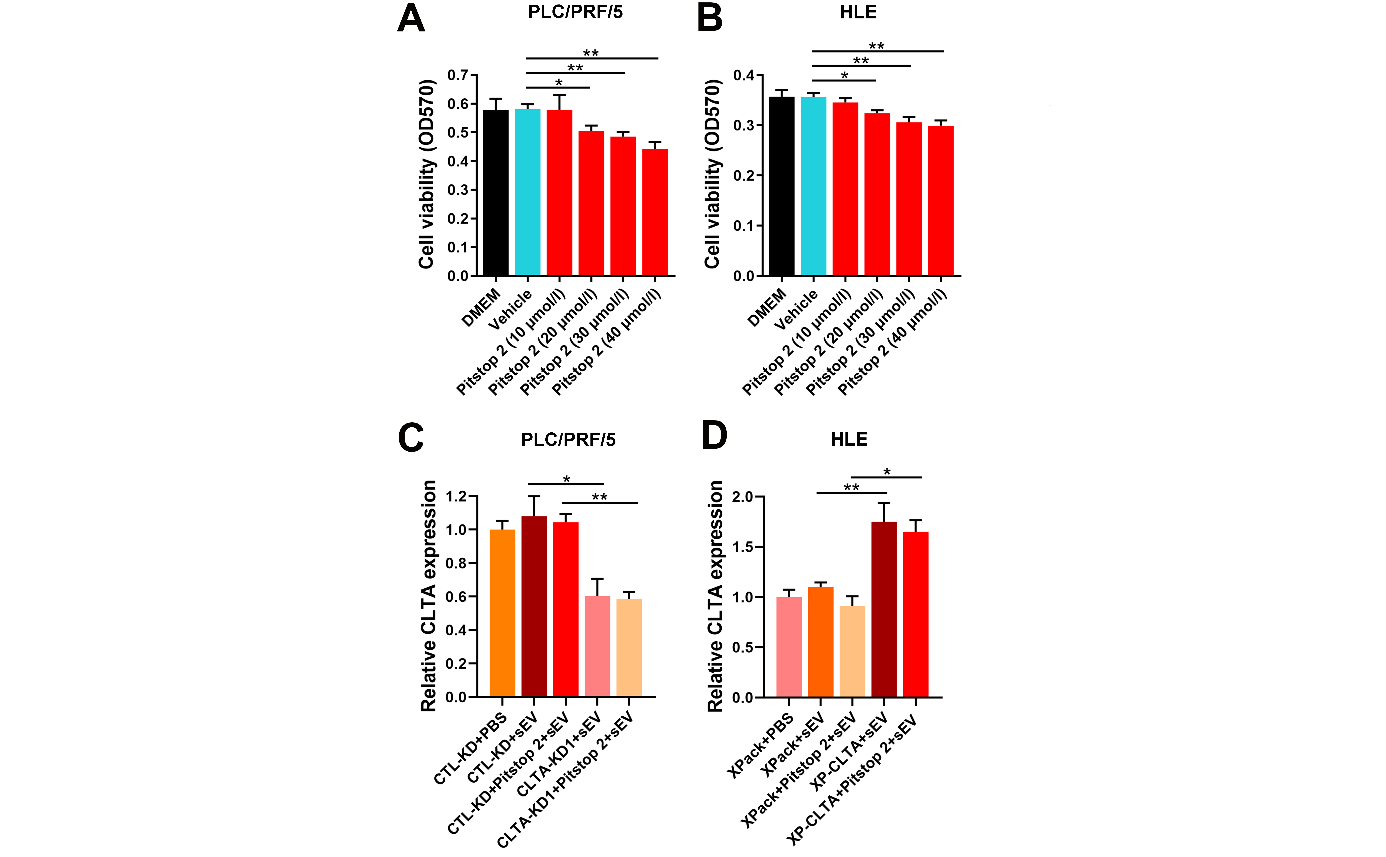


**Fig. S2. Pitstop 2 cannot affect CLTA expression in HCC.** (A) MTT was used to optimize the concentration of Pitstop 2 for PLC/PRF/5 cells. (B) MTT was used to optimize the concentration of Pitstop 2 for HLE cells. (C) Effect of CLTA knockdown and Pitstop 2 on CLTA expression in PLC/PRF/5 cells. (D) Effect of CLTA overexpression and Pitstop 2 on CLTA expression in HLE cells. Data are presented as the mean ± SEM. **p* < 0.05; ***p* < 0.01.

**Supplementary Tables**

**Table S1 The information of cell lines**

| **Name** | **Citation** | **Supplier** | **Cat no.** | **Authentication test method** |
| --- | --- | --- | --- | --- |
| Human liver cell line MIHA | PMID: 10613743 | Provided by Prof. Jayanta Roy-Chowdhury (Albert Einstein College of Medicine, New York) | RRID: CVCL_SA11 | Mycoplasma negative. Validated by both PCR and qPCR. Authenticated by STR. |
| Human HCC cell line Hep3B2.1-7 | PMID: 233137 | ATCC | Cat# HB-8064  RRID: CVCL_0326 | Mycoplasma negative. Validated by both PCR and qPCR. Authenticated by STR. |
| Human HCC cell line HLE | PMID: 52570 | Japanese Collection of Research Bioresources (JCRB, Japan) | Cat# JCRB0404  RRID: CVCL_1281 | Mycoplasma negative. Validated by both PCR and qPCR. Authenticated by STR. |
| Human HCC cell line Huh7 | PMID: 6203805 | Japanese Collection of Research Bioresources (JCRB, Japan) | Cat# JCRB0403  RRID: CVCL_0336 | Mycoplasma negative. Validated by both PCR and qPCR. Authenticated by STR. |
| Human HCC cell line PLC/PRF/5 | PMID: 63998 | ATCC | Cat# CRL-8024  RRID: CVCL_0485 | Mycoplasma negative. Validated by both PCR and qPCR. Authenticated by STR. |
| Human HCC cell line MHCC97H | PMID: 31378681 | Cancer Institute, Fudan University, China | RRID: CVCL_4972 | Mycoplasma negative. Validated by both PCR and qPCR. Authenticated by STR. |
| Human HCC cell line MHCC97L | PMID: 11819844 | Cancer Institute, Fudan University, China | RRID: CVCL_4973 | Mycoplasma negative. Validated by both PCR and qPCR. Authenticated by STR. |
| Human HCC cell line MHCCLM3 | PMID: 12133480 | Cancer Institute, Fudan University, China | RRID: CVCL_6832 | Mycoplasma negative. Validated by both PCR and qPCR. Authenticated by STR. |
| Human embryonal kidney cells 293FT |  | ATCC | PTA-5077  RRID: CVCL_6911 | Mycoplasma negative. Validated by both PCR and qPCR. |

**Table S2 Sequence based reagents**

| **Name** | **Sequence** | **Supplier** |
| --- | --- | --- |
| sh-CLTA-1-F PCR primer | 5’-CCGGGCTGTTGATGGAGTAATGAATCTCGAGATT  CATTACTCCATCAACAGCTTTTTG-3’ | Integrated DNA Technologies, Inc. (IDT) |
| sh-CLTA-1-R PCR primer | 5’-AATTCAAAAAGCTGTTGATGGAGTAATGAATCTCG  AGATTCATTACTCCATCAACAGC-3’ | IDT |
| sh-CLTA-2-F PCR primer | 5’-CCGGGAAGCCTTTGTAAATGACATTCTCGAGAAT  GTCATTTACAAAGGCTTCTTTTTG -3’ | IDT |
| sh-CLTA-2-R PCR primer | 5’-AATTCAAAAAGAAGCCTTTGTAAATGACATTCTCG  AGAATGTCATTTACAAAGGCTTC-3’ | IDT |
| XP-CLTA-F PCR primer | Forward: 5’-CGGAATTCTCATGGCTGAGCTGGATCCG  TT-3’ | IDT |
| XP-CLTA-R PCR primer | Reverse: 5’-GCCTGCAGTCAGTGCACCAGCGGGGCC-3’ | IDT |
| Human-CLTA qPCR primers | Forward: 5’-CGCAGCAAGAGAGCGAGATT-3’  Reverse: 5’-TGGACCATTACTTTCCTGGTAGT-3’ | IDT |
| Human-CLTB qPCR primers | Forward: 5’-CGAGGAGGCTTTCGTGAAGG-3’  Reverse: 5’-GCAGGCGGGACACATCTTT-3’ | IDT |
| Human-CLTC qPCR primers | Forward: 5’-ACGGTTGCTCTTGTTACGGAT-3’  Reverse: 5’-AGGCTAGAATGGCGATCAAAC-3’ | IDT |
| Human-CAPG qPCR primers | Forward: 5’-AGTCAGCATTTCACAAGACCTC-3’  Reverse: 5’-CACCACACCAGGCGAAGAT-3’ | IDT |
| Human-HPRT1 qPCR primers | Forward: 5’-CTTTGCTGACCTGCTGGATT-3’  Reverse: 5’-CTGCATTGTTTTGCCAGTGT-3’ | IDT |
| CAPG-KD siRNA | sc-44920 | Santa Cruz Biotechnology |

**Table S3 Antibodies**

| **Name** | **Supplier** | **Cat no.** | **Clone no.** |
| --- | --- | --- | --- |
| Rabbit polyclonal anti-CLTA | Proteintech | 10852-1-AP | AG1299 |
| Mouse monoclonal anti-CLTA | BioLegend | MMS-423P | 38D12 |
| Rabbit monoclonal anti-CAPG | Abcam | ab181092 | EPR13194 |
| Mouse monoclonal anti-β-actin | Sigma-Aldrich | A5316 | AC-74 |
| Mouse monoclonal anti-EEA1 | Abcam | ab70521 | 1G11 |
| Goat polyclonal anti-Rab11a | St John’s Laboratories | STJ140068 | 8766 |
| Mouse monoclonal anti-Rab7 | Cell Signaling Technology | 95746 | E9O7E |

**Table S4.** **Associations between CLTA mRNA expression and clinicopathological characteristics of HCC patients**

| Clinicopathological | No. of | CLTA mRNA expression | | *p*-value | |  |
| --- | --- | --- | --- | --- | --- | --- |
| characteristics | patients | High (%) | Low (%) | |  | |
| Gender |  |  |  | | 1.000 | |
| Male | 43 | 20 (35.09%) | 23 (40.35%) | |  | |
| Female | 14 | 7 (12.28%) | 7 (12.28%) | |  | |
| Age (years) |  |  |  | | 0.597 | |
| <55 | 26 | 11 (19.30%) | 15 (26.32%) | |  | |
| ≥55 | 31 | 16 (28.07%) | 15 (26.32%) | |  | |
| Microsatellite |  |  |  | | 0.781 | |
| Positive | 24 | 13 (25.00%) | 11 (21.15%) | |  | |
| Negative | 28 | 13 (25.00%) | 15 (28.85%) | |  | |
| Liver cirrhosis |  |  |  | | 0.414 | |
| Present | 25 | 11 (20.75%) | 14 (26.42%) | |  | |
| Absent | 28 | 16 (30.19%) | 12 (22.64%) | |  | |
| Number of tumors |  |  |  | | 0.465 | |
| 1 | 43 | 23 (44.23%) | 20 (38.46%) | |  | |
| >1 | 9 | 3 (5.77%) | 6 (11.54%) | |  | |
| Tumor size |  |  |  | | **0.042** | |
| >5 cm | 36 | 22 (41.51%) | 14 (26.42%) | |  | |
| ≤5 cm | 17 | 5 (9.43%) | 12 (22.64%) | |  | |
| Lymph node metastasis |  |  |  | | 0.561 | |
| Positive | 21 | 12 (25.00%) | 9 (18.75%) | |  | |
| Negative | 27 | 12 (25.00%) | 15 (31.25%) | |  | |
| Vein invasion |  |  |  | | 0.275 | |
| Positive | 25 | 15 (28.30%) | 10 (18.87%) | |  | |
| Negative | 28 | 12 (22.64%) | 16 (30.19%) | |  | |
| TNM stage |  |  |  | | 0.254 | |
| I-II | 18 | 11 (19.64%) | 7 (12.50%) | |  | |
| III-IV | 38 | 16 (28.57%) | 22 (39.29%) | |  | |
| Edmondson-Steiner grade |  |  |  | | 0.577 | |
| 1-2 grade | 23 | 10 (19.23%) | 13 (25.00%) | |  | |
| 3-4 grade | 29 | 16 (30.77%) | 13 (25.00%) | |  | |
| HBV infection |  |  |  | | 0.414 | |
| Positive | 50 | 23 (41.07%) | 27 (48.21%) | |  | |
| Negative | 6 | 4 (7.14%) | 2 (3.57%) | |  | |
| HCV infection |  |  |  | | 0.090 | |
| Positive | 3 | 3 (6.00%) | 0 (0.00%) | |  | |
| Negative | 47 | 20 (40.00%) | 27 (54.00%) | |  | |
| Serum AFP |  |  |  | | 0.779 | |
| >35 ng/ml | 37 | 17 (30.36%) | 20 (35.71%) | |  | |
| ≤35 ng/ml | 19 | 10 (17.86%) | 9 (16.07%) | |  | |

Data in bold indicates statistical significance at *p* < 0.05.

**Table S5. Univariate and multivariate analysis of factors for overall survival in HCC patients**

| Variables | Univariate analysis | | | Multivariate analysis | | |
| --- | --- | --- | --- | --- | --- | --- |
|  | HR | 95% CI | *p*-value | HR | 95% CI | *p*-value |
| Overall Survival | | | | | | |
| Gender  (Male vs. Female) | 0.692 | 0.351-1.364 | 0.288 |  |  |  |
| Age  (≥55 vs. <55) | 1.685 | 0.882-3.216 | 0.114 |  |  |  |
| Microsatellite  (Positive vs. Negative) | 1.805 | 0.946-3.443 | 0.073 |  |  |  |
| Liver cirrhosis  (Present vs. Absent) | 1.449 | 0.770-2.724 | 0.250 |  |  |  |
| Number of tumors  (>1 vs. 1) | 1.328 | 0.553-3.187 | 0.526 |  |  |  |
| Tumor size  (>5 cm vs. ≤5 cm) | 1.137 | 0.575-2.247 | 0.712 |  |  |  |
| Lymph node metastasis  (Positive vs. Negative) | 1.823 | 0.929-3.576 | 0.081 |  |  |  |
| Vein invasion  (Positive vs. Negative) | 1.834 | 0.973-3.457 | 0.061 |  |  |  |
| TNM stage  (III-IV vs. I-II) | 1.967 | 1.032-3.749 | **0.040** | 0.950 | 0.415-2.176 | 0.903 |
| Edmondson-Steiner grade  (3-4 grade vs. 1-2 grade) | 2.279 | 1.171-4.436 | **0.015** | 1.843 | 0.744-4.566 | 0.187 |
| HBV infection  (Positive vs. Negative) | 2.521 | 0.774-8.215 | 0.125 |  |  |  |
| HCV infection  (Positive vs. Negative) | 0.647 | 0.154-2.723 | 0.553 |  |  |  |
| Serum AFP  (>35 ng/ml vs. ≤35 ng/ml) | 2.080 | 1.036-4.178 | **0.040** | 1.513 | 0.571-4.014 | 0.405 |
| CLTA mRNA expression  (High vs. Low) | 2.032 | 1.080-3.824 | **0.028** | 2.100 | 1.028-4.289 | **0.042** |

HR, hazard ratio; 95 % CI, 95 % confidence interval

Data in bold indicates statistical significance at *p* < 0.05.

**Table S6 Univariate and multivariate analysis of factors for disease-free survival in HCC patients**

| Variables | Univariate analysis | | | Multivariate analysis | | |
| --- | --- | --- | --- | --- | --- | --- |
|  | HR | 95% CI | *p*-value | HR | 95% CI | *p*-value |
| Disease-free Survival | | | | | | |
| Gender  (Male vs. Female) | 0.705 | 0.372-1.336 | 0.284 |  |  |  |
| Age  (≥55 vs. <55) | 1.216 | 0.686-2.155 | 0.503 |  |  |  |
| Microsatellite  (Positive vs. Negative) | 1.687 | 0.930-3.061 | 0.085 |  |  |  |
| Liver cirrhosis  (Present vs. Absent) | 1.119 | 0.623-2.010 | 0.706 |  |  |  |
| Number of tumors  (>1 vs. 1) | 1.415 | 0.653-3.066 | 0.379 |  |  |  |
| Tumor size  (>5 cm vs. ≤5 cm) | 1.350 | 0.717-2.542 | 0.353 |  |  |  |
| Lymph node metastasis  (Positive vs. Negative) | 1.742 | 0.926-3.276 | 0.085 |  |  |  |
| Vein invasion  (Positive vs. Negative) | 2.434 | 1.339-4.424 | **0.004** | 1.985 | 0.943-4.179 | 0.071 |
| TNM stage  (III-IV vs. I-II) | 2.593 | 1.406-4.785 | **0.002** | 1.138 | 0.490-2.645 | 0.764 |
| Edmondson-Steiner grade  (3-4 grade vs. 1-2 grade) | 2.387 | 1.290-4.417 | **0.006** | 1.354 | 0.578-3.170 | 0.485 |
| HBV infection  (Positive vs. Negative) | 1.911 | 0.685-5.329 | 0.216 |  |  |  |
| HCV infection  (Positive vs. Negative) | 0.601 | 0.144-2.498 | 0.483 |  |  |  |
| Serum AFP  (>35 ng/ml vs. ≤35 ng/ml) | 2.424 | 1.291-4.551 | **0.006** | 1.538 | 0.685-3.453 | 0.297 |
| CLTA mRNA expression  (High vs. Low) | 1.534 | 0.868-2.714 | 0.141 |  |  |  |

HR, hazard ratio; 95 % CI, 95 % confidence interval

Data in bold indicates statistical significance at *p* < 0.05.

**Supplementary References**

[1] Brown JJ, Parashar B, Moshage H, Tanaka KE, Engelhardt D, Rabbani E, et al. A long-term hepatitis B viremia model generated by transplanting nontumorigenic immortalized human hepatocytes in Rag-2-deficient mice. Hepatology 2000;31:173-181.

[2] Desiere F, Deutsch EW, King NL, Nesvizhskii AI, Mallick P, Eng J, et al. The PeptideAtlas project. Nucleic Acids Res 2006;34:D655-658.

[3] **Tang Z**, **Li C**, Kang B, Gao G, Li C, Zhang Z. GEPIA: a web server for cancer and normal gene expression profiling and interactive analyses. Nucleic Acids Res 2017;45:W98-W102.

[4] **Li T**, **Fu J**, Zeng Z, Cohen D, Li J, Chen Q, et al. TIMER2.0 for analysis of tumor-infiltrating immune cells. Nucleic Acids Res 2020;48:W509-W514.
